# Rhizosphere and Non-Rhizosphere Soil Microbial Communities in Alpine Desertified Grassland Affected by Vegetation Restoration

**DOI:** 10.3390/plants14131925

**Published:** 2025-06-23

**Authors:** Xuan Gao, Hongyu Qian, Rui Huang, Wangyi He, Haodong Jiang, Ao Shen, Zhi Li, Yufu Hu

**Affiliations:** College of Resources, Sichuan Agricultural University, Chengdu 611130, China; gx03250421@163.com (X.G.); qhy1632021@163.com (H.Q.); rhuang0813@foxmail.com (R.H.); hewangyi@stu.sicau.edu.cn (W.H.); jianghaodong@stu.sicau.edu.cn (H.J.); 2024306081@stu.sicau.edu.cn (A.S.); huoyinobi@163.com (Z.L.)

**Keywords:** plant restoration, alpine sandy land, rhizosphere, microbiological characteristics

## Abstract

The rhizosphere serves as a critical interface for plant–soil–microorganism interactions. Rhizosphere soil refers to the soil directly adhering to root surfaces, while non-rhizosphere soil denotes the surrounding soil not in direct contact with roots. This study investigated the characteristics of soil microbial community structure, diversity, and enzyme activity dynamics in both rhizosphere and non-rhizosphere soils of *Salix cupularis* (shrub) across different restoration periods (4, 8, 16, and 24 years) in alpine sandy lands on the eastern Qinghai–Tibet Plateau, with unrestored sandy land as control (CK), while analyzing relationships between soil properties and microbial characteristics. Results demonstrated that with increasing restoration duration, activities of sucrase, urease, alkaline phosphatase, and catalase in *Salix cupularis* rhizosphere showed increasing trends across periods, with rhizosphere enzyme activities consistently exceeding non-rhizosphere levels. Bacterial Chao1 and Shannon indices followed similar patterns to enzyme activities, revealing statistically significant differences between rhizosphere and non-rhizosphere soils after 8 and 24 years of restoration, respectively. Dominant bacterial phyla ranked by relative abundance were Actinobacteria > Proteobacteria > Acidobacteria > Chloroflexi > Gemmatimonadetes. The relative abundance of Actinobacteria exhibited highly significant positive correlations with carbon, nitrogen, phosphorus, and enzyme activity indicators, indicating that *Salix cupularis* restoration promoted improvements in soil physicochemical properties and nutrient accumulation, thereby enhancing bacterial community diversity and increasing Actinobacteria abundance. These findings provide fundamental data for restoration ecology and microbial ecology in alpine ecosystems, offering a scientific basis for optimizing ecological restoration processes and improving recovery efficiency in alpine sandy ecosystems.

## 1. Introduction

The community structure and diversity of soil microorganisms and the extracellular enzymes they secrete directly influence the structure, function, and succession processes of grassland ecosystems [1]. Grassland ecosystems, characterized by relatively low species diversity and ecological instability, exhibit rapid responses in soil microbial community composition and diversity to ecosystem changes [2]. These microbial communities are closely linked to soil functional diversity and play a significant role in soil fertility assessment and ecosystem stability [3]. Due to their high species richness and sensitivity to environmental changes, soil bacteria can adapt by altering their community structure, making their composition and diversity key indicators of grassland ecological stability [4]. Within grassland ecosystems, plants, soil, and microorganisms interact closely, forming a complex organic whole with multifaceted effects [5]. Key microbial groups in grassland soils can positively influence host plant growth by enhancing their adaptability to extreme environments, resistance to pathogenic diseases, and nutrient absorption capabilities [6].

Microbiologist Hiltner defined the rhizosphere as the soil region surrounding plant roots that is influenced by root growth and accumulates abundant nutrients from the soil microenvironment, representing the microzone where plant root activity most intensely and directly affects soil and serving as a critical interface for interactions among plants, soil, and microorganisms [7,8,9], while also being the most active area for material and energy exchange among these three components and playing a vital role in ecosystem nutrient cycling [10]. Plant rhizospheres provide carbon sources and energy for microbial growth and reproduction through the production of low-molecular-weight compounds (root exudates), resulting in rhizosphere soil microorganisms generally exhibiting higher activity and diversity than non-rhizospheric soil microbes (Rhizosphere soil is the soil directly attached to the root system, while non-rhizosphere soil refers to the soil surrounding the roots that is not directly attached) [11]. Simultaneously, rhizospheric microbial metabolism influences root exudate release by modifying the mineral composition of the rhizosphere, root cell membrane permeability, root metabolic activity, and the absorption/utilization of root secretions [12,13]. Despite widespread challenges in grassland ecosystems including desertification, nutrient poverty, and extreme climatic conditions, diverse microbial communities still colonize grassland plant rhizospheres and positively influence plant growth and stress resistance [14]. Meanwhile, the rhizosphere microorganisms in grassland soils contain numerous microbial groups that positively influence plant growth and stress resistance regulation, such as plant growth-promoting rhizobacteria (PGPR) and biocontrol bacteria, among which bacteria represent the most abundant group and hold significant importance for the protection and restoration of grassland ecosystems [4,15]. Precisely for this reason, grassland plant rhizosphere microorganisms have become a current research hotspot. For instance, An et al. [16], using grasslands under different grazing intensities as their research subject, found that grazing significantly reduced the activity of rhizosphere soil microorganisms. Zhang et al. [17] discovered that grazing decreases soil enzyme activity and affects core phyla and genera of rhizosphere bacterial communities, such as Bacteroidota, Rubrobacter, and Gaiella.

Alpine semi-humid grasslands are widely distributed across the eastern margin of the Qinghai–Tibet Plateau, serving as crucial water source areas for both the Yangtze and Yellow Rivers while functioning as a vital ecological barrier with an extremely fragile environment [18]. However, under the combined pressures of climate change and anthropogenic activities, severe grassland degradation has occurred in this region, with localized desertification showing an expanding trend that significantly threatens the ecological integrity, biodiversity, and pastoral economy of the eastern Qinghai–Tibet Plateau [19]. The *Salix cupularis*, a cold and drought-tolerant deciduous shrub from the *Salicaceae* family [20], has been increasingly utilized as a sand-fixing species in ecological restoration projects across alpine sandy regions of the eastern Tibetan Plateau with demonstrated effectiveness [20,21]. Current research has revealed that *Salix cupularis* planting facilitates the reestablishment of surface herbaceous plant communities [20], with Jiang et al. [22] demonstrating its long-term restoration capacity to enhance plant diversity in sandy areas. Hu et al. [23] further investigated the carbon, nitrogen, and phosphorus storage patterns and ecological stoichiometric characteristics in alpine sandy lands with varying planting durations of *Salix cupularis* in the eastern Qinghai–Tibet Plateau. These collective findings substantiate the positive role of *Salix cupularis* in restoring degraded alpine sandy ecosystems, where its rhizosphere’s fertility island effect shows strong correlations with plant age and crown dimensions [24]. Nevertheless, critical knowledge gaps remain regarding the dynamics of soil extracellular enzyme activities, microbial community structure, and diversity associated with rhizosphere nutrient regulation during vegetation restoration processes in these alpine sandy lands.

Therefore, this study focuses on *Salix cupularis* with different planting durations (4, 8, 16, and 24 years) in sandy areas of the semi-humid region on the eastern edge of the Qinghai–Tibet Plateau, investigating the characteristics of soil enzyme activities, microbial community composition and diversity in both rhizosphere and non-rhizosphere soils, while exploring the relationship between soil properties and microbiological characteristics. The research aims to provide a scientific basis for regulating ecological restoration processes and improving rehabilitation efficiency in alpine sandy lands. Three main scientific hypotheses are proposed: (1) Vegetation restoration enhances soil enzyme activities and microbial community diversity; (2) rhizosphere soils exhibit higher enzyme activities and microbial diversity than non-rhizosphere soils; (3) with increasing restoration duration, the fertility island effect of *Salix cupularis* strengthens, and soil enzymes and microorganisms influence soil nutrient supply.

## 2. Results

### 2.1. Characteristics of Soil Enzyme Activity at Different Restoration Years

**Figure 1:**
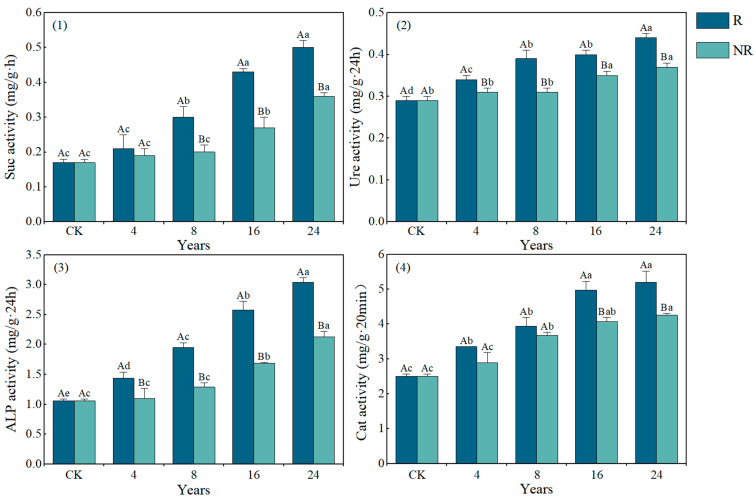
Soil sucrase (**1**), urease (**2**), alkaline phosphatase (**3**), and catalase (**4**) activity at different years of restoration. Note: Different lowercase letters indicate significant differences between years (*p* < 0.05). Different capital letters indicate significant differences between the rhizosphere and non-rhizosphere (*p* < 0.05). R, rhizosphere; NR, non-rhizosphere.

As shown in Figure 1, both rhizosphere and non-rhizosphere soils exhibited increasing trends in sucrase (Suc), urease (Ure), alkaline phosphatase (ALP), and catalase (Cat) activities with prolonged restoration duration (Figure 1). The most substantial increases were observed after 24 years of restoration, with soil Suc, Ure, ALP, and Cat activities showing significant increases of 194.12%, 51.72%, 186.79%, and 107.17%, respectively, compared to CK (*p* < 0.05). Significant differences in these enzyme activities between rhizosphere and non-rhizosphere soils were detected after both 16 and 24 years of restoration (*p* < 0.05). Specifically, after 24 years of restoration, the activities of Suc, Ure, ALP, and Cat in rhizosphere soil were 40.23%, 17.23%, 14.29%, and 22.29% higher than those in non-rhizosphere soil, respectively. These findings clearly indicate that *Salix cupularis* restoration more effectively enhances enzyme activities in rhizosphere soil, with the enhancement effect progressively intensifying as restoration duration increases.

### 2.2. Characteristics of Soil Microbial Diversity at Different Restoration Years

As shown in Table 1, the bacterial α-diversity indices (Chao1 and Shannon) in rhizosphere soil exhibited an initial increase followed by a decreasing trend with extended restoration duration. The rhizosphere Chao1 index was significantly higher than CK after 4, 8, 16 and 24 years of restoration, reaching 1264.67, 1285.72, 1198.80 and 1191.29, respectively, and remained significantly elevated compared to non-rhizosphere soil after 8, 16 and 24 years (*p* < 0.05). Similarly, the rhizosphere Shannon index showed significant increases versus CK at all restoration timepoints (9.49, 9.48, 9.40 and 9.42, respectively), and was significantly higher than non-rhizosphere levels after 24 years (*p* < 0.05). Neither the faith_PD nor Simpson indices demonstrated significant variations with restoration duration or between rhizosphere and non-rhizosphere soils. These results indicate that restoration duration significantly impacts bacterial diversity in alpine sandy lands, with rhizosphere microbial diversity becoming markedly superior to non-rhizosphere communities during advanced restoration stages.

**Figure 2:**
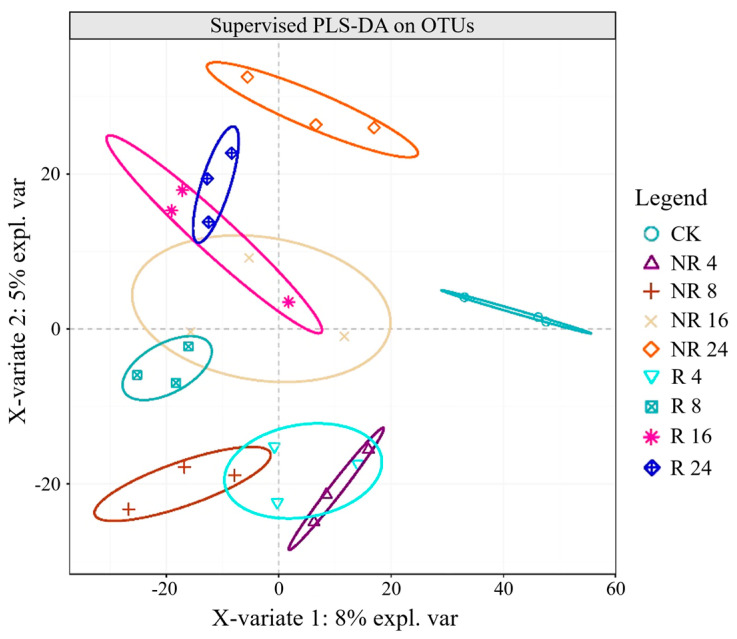
PLS-DA analysis of soil bacteria in the rhizosphere and non-rhizosphere of *Salix cupularis*.

The PLS-DA analysis results indicated that β-diversity exhibited significant clustering effects in rhizosphere remediation after 4 years (R4), non-rhizosphere remediation after 4 years (NR4), and non-rhizosphere remediation after 8 years (NR4); significant clustering effects were also observed in rhizosphere remediation after 8 years (R8), rhizosphere remediation after 16 years (R16), non-rhizosphere remediation after 16 years (NR16), and rhizosphere remediation after 24 years (R24). However, CK showed significant separation effects from other treatments; non-rhizosphere remediation after 24 years (NR24) also exhibited significant separation effects from other treatments, indicating that the duration of remediation had a significant impact on bacterial community structure (Figure 2).

### 2.3. Characteristics of Soil Microbial Community Structure, Composition and Species Abundance at Different Restoration Years

**Figure 3:**
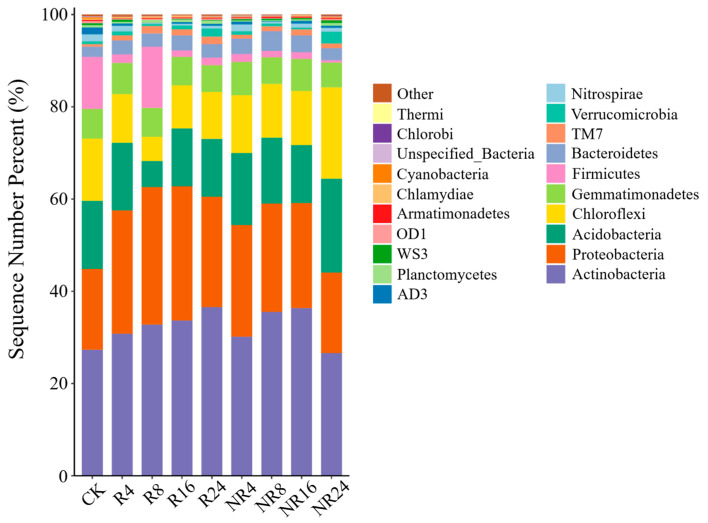
Histogram of species in the classification level of the sample phylum.

The study revealed that the bacterial communities in both rhizosphere and non-rhizosphere soils of *Salix cupularis* across different restoration years were primarily composed of seven dominant phyla (relative abundance >1%). In rhizosphere soils, Actinobacteria accounted for 27.35–36.58%, Proteobacteria 17.52–29.82%, Acidobacteria 5.64–14.72%, Chloroflexi 5.22–13.52%, Gemmatimonadetes 5.81–6.75%, Firmicutes 1.38–13.28%, and Bacteroidetes 2.25–3.29%. Corresponding values in non-rhizosphere soils were as follows: Actinobacteria 26.63–36.37%, Proteobacteria 17.44–24.22%, Acidobacteria 12.57–20.33%, Chloroflexi 11.67–19.80%, Gemmatimonadetes 5.37–7.26%, Firmicutes 0.49–11.28%, and Bacteroidetes 2.25–4.29%. The dominant phyla—Actinobacteria, Proteobacteria, Acidobacteria, Chloroflexi and Gemmatimonadetes—remained consistent between rhizosphere and non-rhizosphere soils without significant alterations (Figure 3).

The results demonstrated that in the rhizosphere, the relative abundance of Actinobacteria showed an increasing trend with restoration duration, reaching 30.82%, 32.87%, 33.70%, and 36.59% at 4, 8, 16, and 24 years, respectively, representing increases of 3.47%, 5.52%, 6.36%, and 9.25% compared to CK (*p* < 0.05). The relative abundance of Proteobacteria in the rhizosphere exhibited an initial increase followed by a decrease with restoration duration, though the changes were not significant (*p* > 0.05). The relative abundance of Acidobacteria significantly decreased to 5.65% after 8 years, representing a 9.07% reduction compared to CK (*p* < 0.05), with subsequent non-significant increases in later restoration stages (*p* > 0.05). Chloroflexi showed a similar trend to Acidobacteria in the rhizosphere. No significant changes were observed in Gemmatimonadetes abundance with restoration duration (*p* > 0.05). In non-rhizosphere soils, Proteobacteria displayed an initial increase followed by a decrease, showing 8.12% and 9.00% increases compared to CK at 8 and 16 years, respectively, followed by a significant decrease at 24 years (*p* < 0.05). The relative abundance of Acidobacteria and Chloroflexi in non-rhizosphere soils significantly increased by 5.6% and 6.28%, respectively, compared to CK after 24 years (*p* < 0.05). No significant changes were detected in non-rhizosphere Gemmatimonadetes abundance across restoration durations (*p* > 0.05).

The restoration with *Salix cupularis* significantly altered the relative abundance of dominant bacterial phyla in alpine sandy soil, creating marked differences between rhizosphere and non-rhizosphere zones. With increasing restoration duration, Actinobacteria showed an increasing trend in the rhizosphere but exhibited an initial rise followed by decline in non-rhizosphere soil, while Acidobacteria and Chloroflexi displayed decreasing trends in the rhizosphere but increasing patterns in non-rhizosphere soil over the restoration period (Table 2).

### 2.4. Correlation Analysis Between Bacterial Communities and Soil Environmental Factors

**Figure 4:**
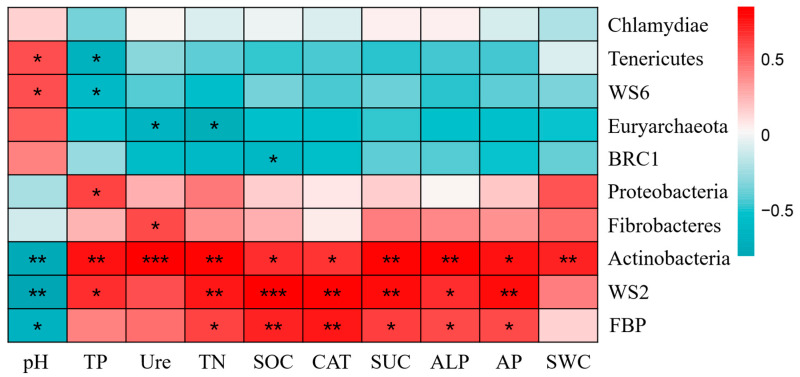
Spearman analysis of soil bacterial phyla and soil environmental factors under *Salix cupularis*. Note: * indicates significant correlation at the 5% level (*p* < 0.05); ** indicates significant correlation at the 1% level (*p* < 0.01); *** indicates significant correlation at the 1‰ level (*p* < 0.001).

The results showed that among the dominant bacterial phyla, Actinobacteria exhibited significant positive correlations with soil SOC and catalase activity (*p* < 0.05), and highly significant positive correlations with soil SWC, TN, TP, AP, sucrase activity, urease activity, and catalase activity (*p* < 0.01). Proteobacteria showed a significant positive correlation with soil TP (*p* < 0.05) (Figure 4).

## 3. Discussion

### 3.1. Effects of Ecological Restoration on Soil Enzyme Activity

Enzymes serve as the primary drivers of soil material transformation and nutrient cycling, playing crucial roles in nutrient mineralization and organic matter decomposition. Soil sucrase is closely associated with the transformation of organic carbon [25]. Numerous studies have investigated differences in enzyme activities between rhizosphere and non-rhizosphere soils across various plant species [26,27]. Research by Zhang et al. [17] and Qiu et al. [28] confirmed that soil enzymes primarily originate from soil microorganisms and plant root exudates, with microbial nutrient supply mainly derived from organic matter in plant residues, demonstrating that enzyme activities are generally higher in rhizosphere than non-rhizosphere soils for most plants [29]. Our findings reveal varying degrees of differences in sucrase, urease, alkaline phosphatase, and catalase activities between rhizosphere and non-rhizosphere soils of *Salix cupularis*-planted sandy lands at the eastern Qinghai–Tibet Plateau across different restoration periods. Enzyme activities showed increasing trends with restoration duration, likely attributable to accumulated litter and decomposed residues from *Salix cupularis* growth improving soil fertility [21], coupled with enhanced windbreak and sand fixation capacity that improved soil water and nutrient retention, reducing nutrient loss while promoting microbial activity and ecological improvement (Table 3). Burke et al. [30] found that vegetation restoration in degraded lands enhances soil enzyme activities through root physiological activities that release enzymes or provide sugars and amino acids via root exudates to nourish rhizosphere microorganisms, consistent with our study results.

Further analysis revealed that the activities of sucrase, urease, alkaline phosphatase, and catalase in the rhizosphere soil of *Salix cupularis* were significantly higher than those in non-rhizosphere soil across different restoration periods, with marked differences between rhizosphere and non-rhizosphere zones. Rhizosphere soil refers to the soil microzone directly adhering to the root surface, which is strongly influenced by root exudates. In contrast, non-rhizosphere soil surrounds the roots but does not make direct contact with them [11]. Therefore, this phenomenon may be attributed to the abundant root systems developed during plant growth, which release diverse enzymes into the soil environment through root exudation [26]. Concurrently, as plants mature, the nutrient enrichment effect in the rhizosphere intensifies, increasing the availability of nutrients in this zone and creating favorable conditions for microbial proliferation. This enhanced microbial activity results in greater microbial biomass and diversity in rhizosphere soil compared to non-rhizosphere soil, ultimately leading to elevated soil enzyme activities [31].

### 3.2. Effects of Ecological Restoration on Soil Microbial Communities

This study found that with increasing restoration duration, the rhizosphere bacterial richness (Chao1 index and Faith_PD index) and diversity (Shannon index and Simpson index) of *Salix cupularis* were consistently higher than in non-rhizosphere soils, with significant differences observed between rhizosphere and non-rhizosphere soils in Chao1 and Shannon indices during later restoration stages. The Chao1 and Shannon indices initially increased and then decreased with restoration time, while Faith_PD and Simpson indices showed no significant changes. This pattern may result from *Salix cupularis* restoration improving soil physicochemical properties, accumulating surface residues, enhancing litter and root decomposition, and increasing nutrient content, which stimulate root exudate formation [31] and positively influence soil microbial diversity, leading to increased richness. During later restoration stages, the establishment of dominant bacterial communities in the rhizosphere may cause bacterial richness to decline and gradually stabilize [32]. These findings align with Shuai et al. [33] research on alpine desertified grassland restoration, suggesting *Salix cupularis* may regulate soil microbial communities. Deng et al. [34] found that Actinobacteria and Proteobacteria consistently dominated the rhizosphere soils of dominant plants in desert grasslands. Similarly, Ding et al. [35] discovered that Proteobacteria, Actinobacteria, and Acidobacteria each exceeded 15% relative abundance in Robinia pseudoacacia rhizosphere and non-rhizosphere soils in the Yellow River Delta, with Actinobacteria significantly more abundant in rhizosphere soils. In our study, Actinobacteria were most abundant in *Salix cupularis* rhizosphere and non-rhizosphere soils, followed by Proteobacteria, indicating microbial community differences between rhizosphere environments while maintaining general soil microbial composition characteristics.

Actinobacteria play the most significant role in cellulose and hemicellulose degradation as well as pectin and lignin transformation [36], with cellulose being a major component of soil organic matter. In this study, the relative abundance of Actinobacteria in rhizosphere soil generally showed an increasing trend with restoration duration and was significantly correlated with soil organic carbon, consistent with previous research findings [33]. This phenomenon may result from the priming effect of organic matter input—plant root exudates and dead roots directly enter the rhizosphere, forming rhizosphere carbon deposits, while Actinobacteria mineralize carbon as an energy source for their own growth and development [37]. Additionally, Actinobacteria possess phosphate-solubilizing capabilities, making their relative abundance indicative of soil phosphorus content [38], which, in our study, also showed a significant positive correlation with soil phosphorus. Proteobacteria can utilize nutrients such as ammonia and methane produced by organic matter decomposition for growth and metabolic activities, and soils with higher organic carbon content favor Proteobacteria growth [38]. In our study, Proteobacteria constituted the second most dominant bacterial phylum in *Salix cupularis* soils after Actinobacteria, showing significant positive correlations with total soil phosphorus and positive associations with soil organic carbon. This occurs because accumulated litter and root exudates from *Salix cupularis* progressively increase soil organic carbon content over restoration time, while Proteobacteria can utilize organic carbon to provide nitrogen fixation capacity for the soil. During early restoration stages, significantly increased carbon and nitrogen content beneath *Salix cupularis* creates favorable nutritional conditions for Proteobacteria growth and metabolism, increasing their abundance. In later restoration stages, when soil nitrogen becomes relatively abundant [21], increased synthesis products may inhibit this bacterial group’s activity, leading to decreased relative abundance of Proteobacteria. As oligotrophic bacteria, Acidobacteria and Chloroflexi showed decreasing trends in the rhizosphere but increasing trends in more nutrient-deficient non-rhizosphere soils, aligning with previous research findings [38].

## 4. Materials and Methods

### 4.1. Site Description

The study area is located in the degraded grassland restoration demonstration zone of Hongyuan County (33°1′ N, 102°37′ E) on the eastern margin of the Qinghai–Tibet Plateau in China [21]. With an average elevation exceeding 3400 m, the region receives an average annual precipitation of 791.95 mm, 60–75% of which is concentrated from May to September, and has a mean annual temperature of 1.1 °C. The soil is classified as cambic arenosol [39], characterized by sandy texture, loose structure, and low nutrient content. The restoration demonstration area features gently undulating mobile, semi-mobile, and semi-fixed dunes with virtually no vegetation cover prior to restoration efforts. Ecological restoration began in the 1980s using *Salix cupularis* as the dominant shrub species, planted as cuttings in holes with row spacing of 1–2 m and plant spacing of 2.5 m. Fencing and grazing prohibition were implemented to reduce sand particle transport and promote stabilization, while grass species such as *Elymus nutans* and *Avena sativa* were interplanted between *Salix cupularis* rows during the initial restoration phase. After successful plant establishment, the sandy lands were left to undergo natural restoration without further human intervention. The initial planting of *Salix cupularis* for this study occurred in 1996, with basic data on *Salix cupularis* across different planting years detailed in Zhou et al. [21]. From 1996 to 2020, both the annual average temperature and precipitation in the study area showed an increasing year-by-year trend [21].

This study adopted a space-for-time substitution approach by selecting naturally growing *Salix cupularis* (4, 8, 16, and 24 years after planting) under enclosed grazing prohibition with relatively uniform environmental conditions as research subjects (see site details in Zhou et al. [21]). For each restoration duration, a 20 m × 20 m plot was randomly established within the *Salix cupularis* growth area, maintaining a minimum distance of 50 m between plots. Three *Salix cupularis* individuals with similar height and crown diameter were selected as experimental replicates within each plot, spaced more than 5 m apart. Unrestored sandy land served as the control (CK). Since no *Salix cupularis* rhizosphere existed in unrestored areas, the CK samples represented both rhizosphere and non-rhizosphere soils. In 25 June 2020, rhizosphere soil was collected using the shaking-off method [40]. The soil samples from 0 to 20 cm depth were excavated using a shovel. After removing the roots, the soil was gently shaken off, and the dislodged soil was collected as non-rhizosphere soil. Subsequently, the soil adhering to the root surface was carefully brushed off using a sterile brush and collected as rhizosphere soil. All samples were then placed into sterile bags for further analysis. Both rhizosphere and non-rhizosphere soils were collected in duplicate: one portion was air-dried and sieved through 0.15 mm mesh for analyzing soil organic carbon (SOC), total nitrogen (TN), total phosphorus (TP), and available phosphorus (AP); the other portion was preserved with dry ice in a 4 °C thermostatic container for measuring pH, soil water content (SWC), activities of sucrase, urease, alkaline phosphatase, catalase, and microbial diversity. The physical and chemical properties of the soil are shown in Table 3.

### 4.2. Measurement Parameters

The moist soil sample was oven-dried at 105 °C for 24 h to measure the SWC. The soil pH (soil:water ratio of 1:2.5) was determined using a combination electrode. SOC was measured via the potassium dichromate external heating method, TN through the semi-micro Kjeldahl method, TP by HClO_4_-H_2_SO_4_ digestion, and AP using 0.5 mol/L pH = 8.5 NaHCO_3_ digestion molybdenum–antimony colorimetric method (Shimadzu Model 721 spectrophotometer, Shanghai, China, 700 nm wavelength) [41]. Soil enzyme activities were determined as follows: sucrase activity by 3,5-dinitrosalicylic acid colorimetry (Shimadzu Model UV-2600 spectrophotometer, Shanghai, China, 508 nm wavelength), urease activity by sodium phenolate-sodium hypochlorite colorimetry (Shimadzu Model UV-2600 spectrophotometer, Shanghai, China, 578 nm wavelength), alkaline phosphatase activity by disodium phenyl phosphate colorimetry (Shimadzu Model UV-2600 spectrophotometer, Shanghai, China, 578 nm wavelength), and catalase activity by ultraviolet absorption spectrometry(Model UV-2600 spectrophotometer, 240 nm wavelength) [42].

Microbial diversity analysis: Total DNA was extracted following the E.Z.N.A.^®^ Soil DNA Kit (Omega Bio-tek, Norcross, GA, USA) instructions, with DNA concentration and purity measured using NanoDrop2000 (Thermo Scientific, Shanghai, China) and extraction quality verified by 1% agarose gel electrophoresis. The V3-V4 hypervariable regions were amplified using primers 338F (5′-ACTCCTACGGGAGGCAGCAG-3′) and 806R (5′-GGACTACHVGGGTWTCTAAT-3′). PCR products were recovered by 2% agarose gel electrophoresis, purified with AxyPrep DNA Gel Extraction Kit (Axygen Biosciences, Union City, CA, USA), eluted with Tris-HCl, and verified by 2% agarose electrophoresis. Quantification was performed using QuantiFluor™-ST (Promega, Madison, WI, USA). Libraries were constructed using Illumina’s TruSeq DNA PCR-Free Library Preparation Kit (Illumina, San Diego, CA, USA), with completed libraries quantified by Qubit and quality-checked before being sequenced on the NovaSeq 6000 platform (Illumina, San Diego, CA, USA).

### 4.3. Statistical Analysis

Statistical analysis was performed using SPSS 20.0 (Chicago, IL, USA). A one-way ANOVA followed by Fisher’s LSD (least significant difference) test was employed to compare differences among treatment means at *p* < 0.05. Spearman’s correlation method was used to analyze relationships between variables.

## 5. Conclusions

This study investigated the characteristics of soil microbial community structure, diversity and enzyme activity dynamics in both rhizosphere and non-rhizosphere soils of *Salix cupularis* shrubs across different restoration periods, while analyzing the relationships between soil properties and microbial characteristics. With increasing restoration duration, the activities of sucrase, urease, alkaline phosphatase, and catalase in *Salix cupularis* rhizosphere showed an increasing trend across all soil layers, with significant differences observed between rhizosphere and non-rhizosphere soils. Rhizosphere enzyme activities were significantly higher than non-rhizosphere levels for all four enzymes. The bacterial Chao1 and Shannon indices generally increased with restoration time and were higher in rhizosphere soils, reaching statistically significant differences between rhizosphere and non-rhizosphere at 8 and 24 years of restoration, respectively. The dominant bacterial phyla in both rhizosphere and non-rhizosphere soils, ranked by relative abundance from high to low, were as follows: *Actinobacteria* > *Proteobacteria* > *Acidobacteria* > *Chloroflexi* > *Gemmatimonadetes*. The relative abundance of soil *Actinobacteria* showed highly significant positive correlations with most carbon, nitrogen, phosphorus and enzyme activity indicators, demonstrating that soil microbial community structure is closely related to soil physicochemical properties and carbon/nitrogen/phosphorus cycling. *Salix cupularis* restoration promoted improvements in soil physicochemical properties along with the cycling, transformation, and accumulation of carbon, nitrogen, and phosphorus, thereby enhancing bacterial community diversity and increasing the relative abundance of *Actinobacteria*. The findings of this study provide fundamental data for restoration ecology and microbial ecology in alpine ecosystems, offering a scientific basis for regulating ecological restoration processes and enhancing recovery efficiency in alpine sandy lands.

## Figures and Tables

**Table 1:** Alpha diversity analysis of *Salix cupularis* rhizosphere and non-rhizosphere bacteria.

Location	Years	Alpha Diversity			
		Chao1	Faith_PD	Shannon	Simpson
R	CK	1034.09 ± 44.69 ^Ab^	96.98 ± 6.96 ^Aa^	8.74 ± 0.41 ^Ab^	0.99 ± 0.01 ^Aa^
	4	1264.67 ± 55.40 ^Aa^	97.32 ± 4.31 ^Aa^	9.49 ± 0.11 ^Aa^	1.00 ± 0.00 ^Aa^
	8	1285.72 ± 14.89 ^Aa^	98.96 ± 3.05 ^Aa^	9.48 ± 0.23 ^Aa^	0.99 ± 0.00 ^Aa^
	16	1198.80 ± 29.93 ^Aa^	89.58 ± 5.33 ^Aa^	9.40 ± 0.08 ^Aa^	1.00 ± 0.00 ^Aa^
	24	1191.29 ± 32.37 ^Aa^	86.38 ± 4.24 ^Aa^	9.42 ± 0.17 ^Aa^	0.99 ± 0.00 ^Aa^
NR	CK	1034.09 ± 44.69 ^Ac^	96.98 ± 6.96 ^Aa^	8.74 ± 0.41 ^Ab^	0.99 ± 0.01 ^Aa^
	4	1193.46 ± 79.46 ^Aa^	95.63 ± 5.26 ^Aa^	9.46 ± 0.06 ^Aa^	1.00 ± 0.00 ^Aa^
	8	1201.40 ± 24.78 ^Ba^	93.14 ± 7.11 ^Aa^	9.46 ± 0.13 ^Aa^	1.00 ± 0.00 ^Aa^
	16	1091.40 ± 55.25 ^Ba^	83.47 ± 2.82 ^Aa^	9.30 ± 0.04 ^Aab^	1.00 ± 0.00 ^Aa^
	24	1115.85 ± 17.87 ^Bb^	86.68 ± 4.87 ^Aa^	9.07 ± 0.18 ^Bab^	1.00 ± 0.00 ^Aa^

Note: Different lowercase letters indicate significant differences between years (*p* < 0.05). Different capital letters indicate significant differences between the rhizosphere and non-rhizosphere (*p* < 0.05). R, rhizosphere; NR, non-rhizosphere.

**Table 2:** Variance analysis of the relative abundance of the main dominant bacteria in the rhizosphere and non-rhizosphere of *Salix cupularis* with different restoration years.

Location	Years	Dominant Bacterial Phyla				
		Actinobacteria	Proteobacteria	Acidobacteria	Chloroflexi	Gemmatimonadetes
R	0	0.27 ± 0.01 ^Ac^	0.18 ± 0.05 ^Aa^	0.15 ± 0.02 ^Aa^	0.14 ± 0.02 ^Aa^	0.06 ± 0.05 ^Aa^
	4	0.31 ± 0.00 ^Ab^	0.27 ± 0.04 ^Aa^	0.15 ± 0.03 ^Aa^	0.11 ± 0.02 ^Aab^	0.07 ± 0.01 ^Aa^
	8	0.33 ± 0.02 ^Ab^	0.30 ± 0.02 ^Aa^	0.06 ± 0.00 ^Bb^	0.05 ± 0.01 ^Bb^	0.06 ± 0.01 ^Aa^
	16	0.34 ± 0.01 ^Ab^	0.29 ± 0.08 ^Aa^	0.13 ± 0.03 ^Aab^	0.13 ± 0.02 ^Aab^	0.06 ± 0.01 ^Aa^
	24	0.37 ± 0.01 ^Aa^	0.24 ± 0.02 ^Aa^	0.13 ± 0.03 ^Bab^	0.10 ± 0.01 ^Bab^	0.06 ± 0.00 ^Aa^
NR	0	0.27 ± 0.01 ^Ab^	0.18 ± 0.05 ^Aa^	0.15 ± 0.02 ^Ab^	0.14 ± 0.02 ^Ab^	0.06 ± 0.05 ^Aa^
	4	0.30 ± 0.05 ^Aab^	0.24 ± 0.04 ^Aa^	0.16 ± 0.00 ^Ab^	0.13 ± 0.02 ^Ab^	0.07 ± 0.02 ^Aa^
	8	0.35 ± 0.03 ^Aa^	0.23 ± 0.04 ^Ba^	0.14 ± 0.04 ^Ab^	0.12 ± 0.03 ^Ab^	0.06 ± 0.00 ^Aa^
	16	0.36 ± 0.02 ^Aa^	0.23 ± 0.02 ^Aa^	0.13 ± 0.01 ^Ab^	0.12 ± 0.02 ^Ab^	0.07 ± 0.01 ^Aa^
	24	0.27 ± 0.02 ^Bb^	0.17 ± 0.03 ^Ba^	0.20 ± 0.01 ^Aa^	0.20 ± 0.01 ^Aa^	0.05 ± 0.01 ^Aa^

Note: Different lowercase letters indicate significant differences between years (*p* < 0.05). Different capital letters indicate significant differences between the rhizosphere and non-rhizosphere (*p* < 0.05). R, rhizosphere; NR, non-rhizosphere.

**Table 3:** Effects of ecological restoration years on soil basic physicochemical properties.

Location	Years	SWC (%)	pH	SOC (g/kg)	TN (g/kg)	TP (g/kg)	AP (mg/kg)
R	0	7.68 ± 0.50 c	7.31 ± 0.04 a	1.15 ± 0.02 d	0.21 ± 0.01 d	146.63 ± 2.79 d	7.94 ± 0.14 d
	4	10.63 ± 0.52 b	6.99 ± 0.04 b	1.37 ± 0.13 cd	0.24 ± 0.00 c	174.94 ± 4.60 c	8.77 ± 0.18 d
	8	11.60 ± 0.84 b	6.86 ± 0.08 b	1.83 ± 0.04 bc	0.25 ± 0.00 bc	185.12 ± 11.83 bc	10.75 ± 0.61 c
	16	11.80 ± 0.75 b	6.64 ± 0.00 c	2.19 ± 0.08 b	0.28 ± 0.00 ab	209.93 ± 11.63 ab	13.54 ± 0.59 b
	24	14.29 ± 0.89 a	6.46 ± 0.08 c	2.78 ± 0.37 a	0.30 ± 0.00 a	231.27 ± 5.64 a	16.71 ± 0.76 a
NR	0	7.68 ± 0.50 b	7.31 ± 0.04 a	1.15 ± 0.02 b	0.21 ± 0.01 b	146.63 ± 2.79 c	7.94 ± 0.14 c
	4	9.43 ± 1.56 a	7.03 ± 0.05 b	1.21 ± 0.10 b	0.21 ± 0.01 b	158.39 ± 4.40 b	8.12 ± 0.43 c
	8	10.91 ± 2.35 a	6.98 ± 0.02 ab	1.34 ± 0.14 b	0.22 ± 0.01 ab	176.60 ± 15.53 ab	9.75 ± 1.16 bc
	16	11.24 ± 0.52 a	6.94 ± 0.06 ab	1.83 ± 0.04 a	0.24 ± 0.01 ab	193.25 ± 4.53 a	11.51 ± 0.39 ab
	24	12.21 ± 0.65 a	6.88 ± 0.03 c	2.00 ± 0.05 a	0.24 ± 0.00 a	190.77 ± 13.65 a	13.70 ± 0.90 a

Note: SWC, soil water content; SOC, soil organic carbon; TN, total nitrogen; TP, total phosphorus; AP, available phosphorus. Different lowercase letters indicate significant differences between years (*p* < 0.05). R, rhizosphere; NR, non-rhizosphere.

## Data Availability

The original contributions presented in this study are included in the article. Further inquiries can be directed to the corresponding author(s).
